# Learning and Use of eHealth Among Older Adults Living at Home in Rural and Nonrural Settings: Systematic Review

**DOI:** 10.2196/23804

**Published:** 2021-12-02

**Authors:** Ella Airola

**Affiliations:** 1 Media Education Hub Faculty of Education University of Lapland Rovaniemi Finland

**Keywords:** aged, barrier, digital competence, deinstitutionalization, eHealth, home care, learning, older adult, rural health

## Abstract

**Background:**

Care policies emphasize deinstitutionalization and *aging in place* in response to demographic changes. Different eHealth technologies are one way to achieve this aim. However, there is a need to better understand older adults’ needs for eHealth services, and thus, these health solutions require further exploration.

**Objective:**

The purpose of this systematic literature review is to appraise, synthesize, and summarize the literature on older adults’ (aged ≥60 years) eHealth learning and use in real home settings, particularly in rural and remote areas, with a focus on the social and cultural context.

**Methods:**

A systematic search was conducted in January 2020 using 4 academic databases. The studies by means of qualitative thematic analysis to identify the barriers, enablers, and support practices involved in the domestication process were examined. In addition, we identified the various meanings attached to eHealth technologies for older adults living in rural and remote areas.

**Results:**

In total, 31 empirical studies published between 2010 and 2020 were included in this review. A total of 17 articles included participants from rural and remote areas. The most regularly reported barriers related to older adults’ learning to use and use of eHealth were health-related difficulties, such as cognitive impairment or impaired hearing. The most reported enabler was the support provided for older adults in learning and use of eHealth. Support mainly comprised older adults’ own digital competences, which were distributed with their social network. It was found that eHealth technology is needed for rural and remote areas to facilitate access and reduce logistical barriers to health care services.

**Conclusions:**

The literature review provided information and practical implications for designers, health care providers, and policy makers. On the basis of these findings, eHealth technologies should be easy to use, and adequate support should be provided to older adults for use.

## Introduction

### Background

The aim of this systematic literature review is to advance the understanding of older adults’ (aged ≥60 years) eHealth learning and use during its domestication. The main target is older adults living in rural and remote environments, as they are far removed from traditional health care services [1-4]. According to the World Health Organization, health is understood as “a state of complete physical, mental, and social well-being and not merely the absence of disease of infirmity” [5]. As a general practice, eHealth can be seen as an umbrella concept for different health care services delivered or improved through information and communication technologies (ICTs) [6,7].

On the basis of future demographic changes, the world will face two challenges: a growing care burden per capita and organizational changes to health care systems [8,9]. To achieve its sustainable development goals, the United Nations [8] has outlined key policy changes that deal with current and future population dynamics. Recommendations include investing in life-long learning, especially digital competences, and promoting healthy aging and long-term care systems to meet the needs of the aging population. Care policy throughout Europe has emphasized deinstitutionalization and *aging in place* to reduce paternalistic care and improve the quality of care [10,11]. Furthermore, previous research in many cultural settings has found that older adults prefer living at home for as long as possible [9].

### Theoretical Framework

This study’s theoretical framework is built on the concept of *distributed and situated digital competence*, *technology domestication,* and previous research on *barriers and enablers* of technology use. The European Commission’s *DigComp 2.0: The Digital Competence Framework for Citizens* [12] defines digital competence as a combination of knowledge, skills, and attitudes related to the use of ICT tools. One competence is the protection of health and well-being, which means, for example, being aware of digital technologies that can be used to enhance social well-being and social inclusion. However, the framework can be criticized for being decontextualized and centered around individuals. Therefore, a socially and culturally oriented approach is followed, and digital competences are understood as the distributed and situated competences of older adults and their social networks [13,14].

The concept of domestication focuses on how technology users and nonusers adopt technologies culturally and socially in their everyday lives [15-17]. The domestication of technology includes four dimensions: appropriation, objectification, incorporation, and conversion. The process starts with appropriation, which does or does not create a relationship with the new technology, and continues to objectification, which is when the technology is given a place at home. Incorporation focuses on the technology’s place in and influence over the user’s everyday routines. Finally, the technology becomes familiar, and conversion is achieved [15-21]. Central to domestication are the public and personal meanings attached to the technology, which actively transform during the process [19]. Meanings are understood as individuals’ thoughts and feelings regarding the technology and how it is seen as part of his or her own cultural context [22,23].

Research into health-related barriers has found that the key reasons older adults avoid ICT adoption are the accessibility of support, their health status, lack of need or interest, functionality, added value, cost, and concerns regarding privacy and trust [24,25]. In the present review, barriers were not understood as insurmountable obstacles but rather as challenges that can be solved. Conversely, the following key enablers influence older adults’ use of eHealth: motivation, support, and feedback [26]. In addition, meaningful function and aesthetics are important when older adults adopt technology [27].

### Objective

Recent international literature reviews on eHealth and older adults focus on a variety of topics: eHealth access and use from a health equity perspective [28], the facilitators of and barriers to eHealth use [24,26,29], and user involvement in technology design, including eHealth technologies [30,31]. In addition, Cheng et al [32] published a systematic review of eHealth interventions targeted at socially disadvantaged groups, including older adults. However, we still lack a review that examines older adults’ eHealth learning and use in real home settings and in a rural context from users’ perspectives. The purpose of the present review is to fill this gap by focusing on studies that report barriers and enablers that older adults face when learning to use and while using eHealth technology and how older adults are supported during the process. In addition, what eHealth technology means to older adults in rural and remote areas was examined. In reviewing relevant studies, this research seeks to answer the following questions:

1. What barriers and enablers are related to the learning and use of eHealth technologies in domestication processes among older adults living at home?
2. How are older adults living at home supported in their domestication of eHealth technologies?
3. What are the meanings attached to eHealth technologies for older adults living in rural and remote areas?

## Methods

### Overview

**Figure 1:**
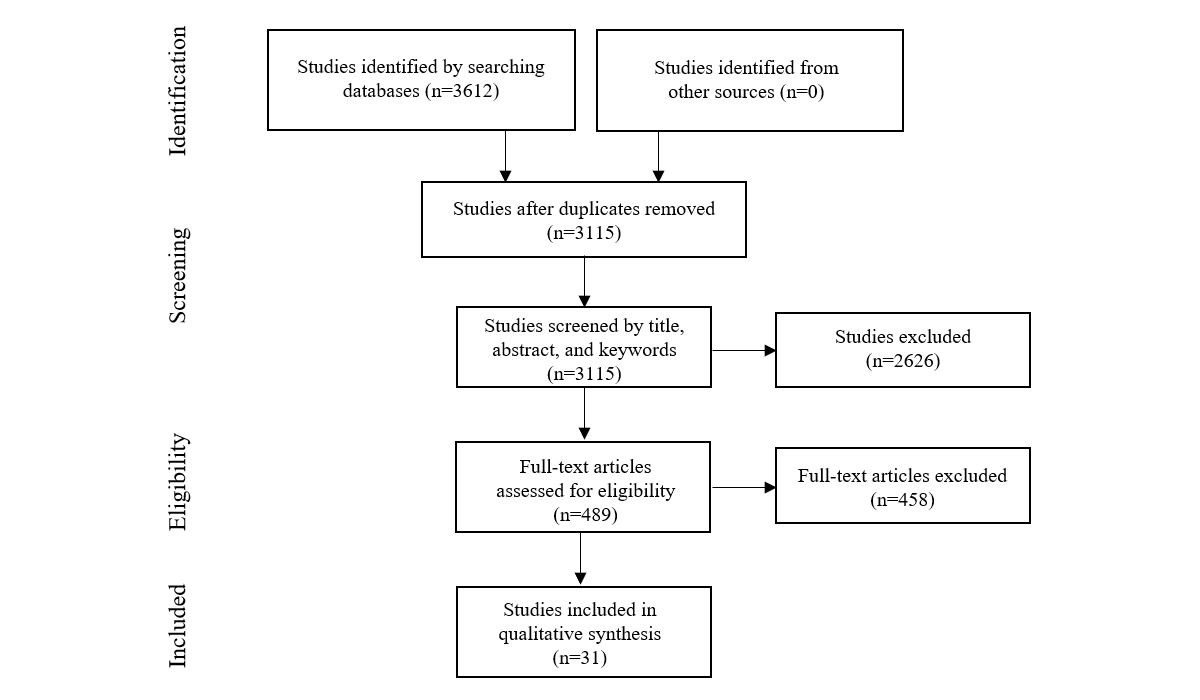
Flow of information through the different phases of the systematic review.

The methodology follows the standard PRISMA (Preferred Reporting Items for Systematic Reviews and Meta-Analyses) guidelines for studies that evaluate health care research [33]. PRISMA includes a 27-item checklist (Multimedia Appendix 1 [33]) and a 4-phase diagram (Figure 1) to help authors ensure clarity and transparency. A systematic literature review was selected “to appraise, synthesize, and summarize” [34] the literature and determine whether further primary research in the area is needed. The entire review process was undertaken by 1 author, and there were no additional authors.

### Search Strategy

To begin the search process, the author consulted an information specialist at her university library regarding search phrases and relevant databases. A literature search was conducted in January 2020 and included the following international web-based databases: ProQuest (ERIC—Education Collection, Social Science Database, Applied Social Sciences Index and Abstracts, Sociological Abstracts, and Sociology Database), Ebsco (AgeLine, Academic Search Elite, and CINAHL Complete), Web of Science (Arts and Humanities Citation Index, Social Sciences Citation Index, Science Citation Index Expanded, and Emerging Sources Citation Index), and Scopus (Elsevier). Relevant articles were found using the search terms specified in Textbox 1 and their combinations. An example search string used to search Scopus (Elsevier) can be found in Multimedia Appendix 2. The search was applied to the field title, abstract, and keywords. Although the conversions around eHealth began at the turn of the millennium [6], the review’s start date of 2010 was chosen as there has been a lot of change in health technologies over the past decade [35]. The initial database search yielded 3612 papers (n=393, 10.88% from ProQuest, n=394, 10.91% from Ebsco, n=2767, 76.61% from Web of Science, and n=58, 1.61% from Scopus). No articles were included that were identified from sources other than the abovementioned databases.

Content and search terms of literature search. During the search, some terms were truncated using “*” so the search considers all the results including the first part of the word. For example, by searching learn*, the search also considers terms *learner* and *learning*.

**Content and search terms**

- Older people:* older, senior, elderly, aged people, old age user, elder*
- Rural: *rural, remote, sparsely populated area*
- Use: education, learn*, competence, digital skill, geragogy, use, reject, active aging, adoption, acceptance, barrier, enabler, facilitator
- Digital technology: online, ICT, information, computer, internet, electronic, techolog*, digital, smart, management tool, virtual, mobile, robot, tele*, monitoring, assist*, gerontechology, compliance, reminder, dispens*, video, application, device
- Health: *health, care, wellbeing, physical, mental, social*
- Home: *aging in place, independent, home, everyday life, living, daily life, domestication*

### Study Selection

Figure 1 shows a flowchart depicting how PRISMA was used to select the studies. All articles were exported to Refworks (ProQuest), a reference management system that automatically discards duplicate papers. Of the 3612 papers, the removal reduced the number of papers to 3115 (86.24%). The remaining articles were screened for title, abstract, and keywords to select studies relevant to the research topic. Following the screening of the 3115 articles, a total of 489 (15.7%) articles were obtained. The remaining articles were exported to NVivo 12 (QSR International), a qualitative data analysis software, and the remaining duplicates were manually discarded. Full-text articles were then assessed for eligibility based on the preliminary inclusion and exclusion criteria.

Finally, full-text articles (31/489, 6.3%) that met the following criteria (Textbox 2) were accepted: published in a peer-reviewed scientific journal; written in English; published between January 1, 2010, and January 22, 2020; had an empirical study design aimed at supporting older adults’ use of eHealth technology in real home settings at the user’s home; completely or partially focused on technology users’ perspectives; had participants with a mean age of 60 years; and included participants who were older adults with or without health conditions. Initially, the aim was to select studies that included only older adults aged >65 years, which is a common age limit for older adults in research [36]; however, because of the low number of studies, the age range was extended to a mean of 60 years. In addition, living in rural or remote areas was a planned inclusion criterion; however, it was modified to living in any area, as not enough studies met the original criterion. However, one research question focused only on studies conducted in rural and remote areas.

**Inclusion and exclusion criteria.**

**Inclusion criteria**

- Academic paper published in a peer-reviewed scientific journal
- Written in English
- Published between 2010 and 2020
- Had an empirical study design aimed at supporting older adults’ eHealth use
- Was located in real home settings at the user’s home
- Had participants with a mean age of 60 years
- Completely or partially focused on users’ perspectives

**Exclusion criteria**

- Did not include older adult participants
- Was not conducted in real home settings
- Was conducted in a laboratory or included no user experience with eHealth
- Was a review or theoretical study
- Only had a biomedical or technical perspective

### Data Exclusion and Analysis

For the final set of full-text articles, a data extraction sheet was developed (Multimedia Appendix 3 [37-65]). The gathered data included information on article titles, authors, journals, publication years, methods, eHealth technologies, target groups, total number of participants, participant genders and ages, participants’ area of residence (eg, rural or remote), and countries of residence. The included articles were analyzed in NVivo 12 using a qualitative thematic approach guided by the concepts of domestication and digital competence. In addition, previous research on the barriers to and enablers of technology use was used. The author thoroughly read and reread the articles and identified 154 subcategories that described significant sentences and phrases concerning the research questions. The obtained subcategories were clustered into 8 upper-level categories to produce a more nuanced understanding.

## Results

### Study Characteristics

The selected 31 articles were published in 29 different journals representing multidisciplinary research. Of the 31 papers, 16 (52%) were quantitative, 4 (13%) were qualitative, and 11 (35%) were mixed methods studies. Most studies used >1 method; only 16% (5/31) used just one method. The most common qualitative data collection methods were surveys and questionnaires (20/31, 65%) and technical logs (12/31, 39%). The most common qualitative data collection methods were interviews (9/31, 29%) and focus groups (5/31, 16%). The studies were conducted in the following 12 countries: the United States (9/31, 29%), Canada (2/31, 6%), Italy (2/31, 6%), Spain (2/31, 6%), Sweden (2/31, 6%), the United Kingdom (2/31, 6%), France (1/31, 3%), Germany (1/31, 3%), Ireland (1/31, 3%), Lebanon (1/31, 3%), Norway (1/31, 3%), and Switzerland *(*1/31, 3%). In 19% (6/31) of studies, the country was not identified.

The number of participants ranged from 1 to 4380, although not all were older adults. In all articles, either the mean or median age and SD or the age range was defined. However, in studies in which only the SD and mean or median age were given, it was not possible to strictly count the age of the youngest and oldest participants. In 90% (28/31) of articles, the mean or median age of the technology users was ≥60 years, and in 39% (12/31) of articles, the minimum age was >60 years. Some articles (6/31, 19%) included technology users aged<50 years, and it was possible to separate them from the older adults in the findings. This means that the findings were still based on participants who were aged >60 years or whose mean or median age was ≥60 years. In 42% (13/31) of articles, most participants were women, and in 35% (11/31) of articles, most participants were men. In 6% (2/31) of papers, female and male participants were equally represented, and in 16% (5/31) of papers, gender was not defined.

All participants used eHealth technologies in real home settings. The eHealth technologies that occurred most frequently were mobile apps (18/31, 58%), monitoring systems (12/31, 39%), web-based platforms (11/31, 35%), assistive technologies (2/31, 6%), and ambient awareness technology (1/31, 3%). No eHealth robots were included in this study. Most technologies were targeted at older adults with different noncommunicable diseases. Of those, the largest target groups were cardiac disease (5/31, 16%), diabetes (3/31, 10%), and cancer (3/31, 10%). In total, 2 technologies were used for older adults with polypharmacy issues and 2 for those in palliative care. In 16% (5/31) of studies, eHealth technologies were aimed at isolated older adults, and in 13% (4/31) of studies, the technology was for older adults with depression, anxiety, or apathetic qualities. In total, 1 eHealth technology was only for healthy older adults, and 5 were for older adults or adults of any age group in general. In some studies, the participants were veterans (4/31, 13%), and in others, they were older family caregivers (4/31, 13%).

### Barriers and Enablers in the Domestication Process

#### Overview

The first research question focused on the barriers and enablers related to the learning and use of eHealth technologies in the domestication process among older adults living at home. Thematic analysis produced 111 subcategories related to these barriers and enablers. The subcategories were clustered into 4 dimensions of the domestication process [15,17]: appropriation (40/111, 36%), objectification (10/111, 9%), incorporation (27/111, 24.3%), and conversion (34/111, 30.6%). A summary of these findings is presented in Tables 1 and 2.

**Table 1:** Summary of the findings concerning the barriers to learning and use of eHealth technologies among older adults (N=31).

Dimension of domestication and barriers				Papers, n (%)
**Appropriation**				
	**eHealth technology**			
		Lack of connectivity	9 (29)	
		Technical problems	8 (26)	
		Difficult to use	6 (19)	
		Unclear instructions	5 (16)	
		Cost	4 (13)	
		Technical limitations	4 (13)	
		Difficult to learn to use	3 (10)	
		No feedback	2 (6)	
		Lack of effectiveness	1 (3)	
		Lack of technical device	1 (3)	
		Technology was unexpected	1 (3)	
	**Old age user**			
		Health-related difficulties	17 (55)	
		Lack of previous experience	5 (16)	
		Uncertain with the technology	5 (16)	
		Irritation or frustration	4 (13)	
		Lack of motivation or interest	2 (6)	
		Personal factors	2 (6)	
		Fatigue	2 (6)	
		Being intimidated	1 (3)	
		Lack of digital competence	1 (3)	
		Skeptical	1 (3)	
		Unwilling to use the technology	1 (3)	
**Objectification**				
	**Object at home**			
		Design	2 (6)	
		Placement in the home	2 (6)	
		Ergonomics	1 (3)	
	**Data protection and security of the eHealth**			
		Concerns about security or privacy	4 (13)	
		Lack of reliability	2 (6)	
**Incorporation**				
	**Everyday life**			
		Unsuitable for everyday life	3 (10)	
		Time constraints or power dynamics	3 (10)	
		Lack of utility	2 (6)	
		Inappropriate technology	1 (3)	
		Logistical difficulties	1 (3)	
		Not meaningful information	1 (3)	
		Not meaningful service	1 (3)	
		Technology used only occasionally	1 (3)	
**Conversion**				
	**Social interactions**			
		Need for face-to-face contact	7 (23)	
		Lack of support	3 (10)	
		Family relationships	2 (6)	
		Feeling like an outsider	1 (3)	
		Lack of communication	1 (3)	
		Lack of patient–professional communication	1 (3)	
		Shyness	1 (3)	
		unable to use independently	1 (3)	
	**Society and culture**			
		Older age	4 (13)	
		Not culturally relevant	3 (10)	
		Living in rural area	2 (6)	
		Lower socioeconomic status	1 (3)	
		Being female	1 (3)	

**Table 2:** Summary of the findings concerning the enablers of learning and use of eHealth technologies among older adults (N=31).

Dimension of domestication and enablers				Papers, n (%)
**Appropriation**				
	**eHealth technology**			
		Usability	18 (58)	
		Personalization or flexibility	13 (42)	
		Familiarity	9 (29)	
		Feedback	7 (23)	
		Accessibility	3 (10)	
		Automated service	1 (3)	
		Novelty effect	1 (3)	
		Offers personal challenge	1 (3)	
		Positive experiences of others	1 (3)	
	**Old age user**			
		Satisfaction	14 (15)	
		Confidence or self-esteem	4 (13)	
		Self-efficacy	4 (13)	
		Feeling of success	3 (10)	
		Open-minded	3 (10)	
		Digital competence of the user	2 (6)	
		Interest in electronic devices	1 (3)	
		No feeling of privacy loss	1 (3)	
		Own choice	1 (3)	
**Objectification**				
	**Object at home**			
		Placement in the home	2 (6)	
		Design	1 (3)	
	**Data protection and security of the eHealth**			
		Technology’s security or safety	2 (6)	
		User privacy	2 (6)	
		Reliability	1 (3)	
**Incorporation**				
	**Everyday life**			
		Suitable for everyday life	10 (32)	
		Service provided educational information	8 (26)	
		Active part of user’s own care, self-care	7 (23)	
		Playful	7 (23)	
		No logistical barriers	7 (23)	
		No temporal barriers	6 (19)	
		Improve quality of daily life	6 (19)	
		Useful service	6 (19)	
		Increased security	5 (16)	
		Systematic use of the technology	4 (13)	
		Extends one’s own habitat	3 (10)	
		Healthier lifestyle	3 (10)	
		Meaningful service	3 (10)	
		No financial barriers	3 (10)	
		Brings joy	2 (6)	
		Fewer clinical visits	2 (6)	
		Technology provides freedom	2 (6)	
		Helpful	1 (3)	
		Not in a hurry	1 (3)	
**Conversion**				
	**Social interactions**			
		Support practices	27 (87)	
		Social connectedness and belonging	10 (32)	
		Patient–professional communication	9 (29)	
		Visual contact	4 (13)	
		Shared experience	3 (10)	
		Face-to-face meeting	2 (6)	
		Group assignments	2 (6)	
		Social comparison	2 (6)	
		Feeling less lonely	1 (3)	
		Individual attention	1 (3)	
		Loneliness	1 (3)	
		Relationship with technology	1 (3)	
	**Society and culture**			
		Cost-effectiveness	8 (26)	
		Cultural relevance	4 (13)	
		Supports independence	2 (6)	
		Feeling of being equal	2 (6)	
		Being female	2 (6)	
		User feels like an active citizen	1 (3)	
		Being considered *cool*	1 (3)	
		Educated or employed	1 (3)	
		Older age	1 (3)	

#### Appropriation

Of the 31 articles, 30 (97%) reported barriers and enablers in the appropriation phase of the domestication process; this dimension had more barriers than the others. Older users’ *health-related difficulties* was the most common barrier in the appropriation phase and the whole domestication process (17/31, 55%). For example, difficulties included cognitive impairment or dementia [37-39] and impaired hearing or vision [40,41]. The most common barriers related to the eHealth technologies themselves were *a lack of connectivity* (eg, a lack of access to fast enough internet connections), which was reported in 29% (9/31) of articles [42,43] and other *technical problems*, which were reported in 26% (8/31) of articles [44,45]. *The usability* of eHealth technology, which included ease of use, was the ruling enabler and was reported in 58% (18/31) of studies [46,47]. *Satisfaction* was also seen as a significant enabler in the domestication process and was reported in 45% (14/31) of studies [53,66]. In addition, *personalization or flexibility* of eHealth technology was reported as an enabler in 42% (13/31) of studies [48,49]. That the technology o*ffered a personal challenge* was reported as an enabler in 3% (1/31) of article [37]; the article reported that if the tasks proposed in a mental training system are slightly above users’ capabilities, users’ motivation is increased and training outcomes are improved. That the *technology was unexpected* was reported as a barrier in 3% (1/31) of articles [51], and this refers to users feeling they had received a remote monitoring system unexpectedly without a choice and without knowing what the system was.

#### Objectification

Objectification was the least-coded dimension, and there was not a big difference between the different subcategories. The *design* of the eHealth technology and its placement in the home were reported as barriers and enablers. As a barrier, design referred to the look of a computer [50] and the weight and size of a remote monitoring system [51]. As an enabler, it was connected to the range of soundscapes provided to the users in an interaction radio [52]. In 3% (1/31) of articles [50], it was found that if a technology is placed *out of the way*, the placement can be a barrier to its use; however, if it is placed where the user spends time, it is used more often. In the Ottenberg et al [51] study, in addition to the weight and size of the system, wiring configurations challenged its placement. Furthermore, *ergonomic challenges*, such as typing or using a mouse, were reported as barriers in 3% (1/31) of articles [40], and this was in the context of a web-based pain management program designed for older adults with chronic pain. The objectification dimension also included barriers and enablers related to data protection and security. Here, the most common barriers were *concerns about security or privacy* [39,51,53,54]. The leading enablers were *technology security or safety* [38,51] and *user privacy* [45,50].

#### Incorporation

Of the 31 articles, 24 (77%) reported barriers and enablers during the incorporation phase. As reported in 32% (10/31) of articles, the most common enabler was that the eHealth technology was *suitable for everyday life* of older adults. For example, this was evident in how users did not actively think they used the technology [52], were able to use it in a self-selected environment [38,53], and found that it matched their needs [55,56]. A total of 2 equally common barriers, each reported in 10% (3/31) of articles, were that the technology was *unsuitable for everyday life* and that there were *time constraints or power dynamics* in using it, which means, for example, that users had scheduling conflicts [38], were busy [41,53], or expected faster replies via the technology [53]. The reasons the technology was not suitable for users’ daily lives were that the users had to change or constrain their behaviors when using it [52], were busy or on holiday [53], and had experienced a stressful life event that caused them to disengage with the service [56].

#### Conversion

The conversion dimension had more enablers than the other dimensions. The most common enabler in the conversion phase and the entire domestication process was *support practices*, which was reported in 87% (27/31) of articles. The findings are presented in detail in *the Support Practices During Domestication* section below. Regarding social interactions, s*ocial connectedness and belonging* were mentioned as enablers in the learning and use of eHealth technology in 32% (10/31) of studies [44,57]. The common barriers related to social interactions were that older adults had a *need for face-to-face contact* [39,51] and felt a *lack of support* while using the technology [44,51,58].

Regarding society and culture, *cost-effectiveness* was the most common enabler in the conversion phase, as reported in 26% (8/31) of articles. However, it was almost never reported from a user’s perspective and was more society driven, providing “huge savings in clinical costs” [59], “significant saves in the health budgets” [60], and “suggesting a cost reduction for the health care system” [46]. In addition, in 13% (4/31) of articles, *cultural relevance* was noted as an enabler, which means, for example, that the users could incorporate their religious or spiritual perspectives into the service [48] or that televisions were used as devices, as they were best integrated into the older adults’ lives [37,59].

In addition, o*lder age* and *being female* were reported as enablers and barriers in different articles. For example, 3% (1/31) of articles [60] reported that women needed more assistance using a mobile health app than men, and in 6% (2/31) of articles [53,67], women were more active eHealth technology users than men. Older age was mainly seen as a barrier in cases where older adults needed more help using the technology [60] or used it less than younger people [60-62,67]. However, in 3% (1/31) of articles [60], older patients reported greater improvements in several measures than younger patients. In 3% (1/31) of articles [62], it was reported that *a* l*ower social class* reduced the prevalence of eHealth technology use.

### Support Practices During Domestication

To understand how older adults were supported while domesticating eHealth technologies, the thematic analysis produced 25 subcategories related to their learning to use and the use of technology. The subcategories were clustered into two upper-level categories: social network support (22/31, 71%) and nonsocial support (3/31, 10%). Of the 31 articles, 27 (87%) reported that support was provided to users during the domestication process [15,17].

#### Social Network Support

Social network support was provided in 87% (27/31) of articles. During the learning process at the beginning of domestication, *face-to-face support* was more common than long-distance support. Face-to-face support was provided to users in 45% (14/31) of articles and long-distance support in 13% (4/31) of articles. The most common way to teach older adults was *a training session*, which was used in 42% (13/31) of articles. Training sessions were used to introduce the users to the use of the technology, and it could happen either at users’ homes [45,47] or in a clinical setting [38,47]. The training was usually given by technical staff [59], health care professionals [49,63], or the researcher of the study [53]. *Long-distance support* was more common than face-to-face support after initial training. Long-distance support was provided to users in 39% (12/31) of articles and face-to-face support in 10% (3/31) of articles. Long-distance support was mostly provided *via phone*, and it was mainly related to technical issues [59] or questions related to the study [61], although it also took the form of consultation with a health care professional [42] or communication with a peer [38]. In some articles, it was not possible to identify whether social network support was face-to-face or long-distance.

During the domestication process, eHealth technology users distributed their digital competence [13,14] with the following members of social networks: *spouses, grandchildren, other family members and relatives, family caregivers, peers, friends, health care professionals, coaches, researchers, technical staff,* and *proctors*. Social networks expanded as the domestication process went further. Digital competences were most commonly distributed among health care professionals, such as nurses, therapists, and social workers. This was reported in 48% (15/31) of articles [42,44]. Second, technical staff was mentioned in 23% (7/31) of studies [39,63].

#### Nonsocial Support

Nonsocial support practices in the learning process were *written instructions* [37,47,49,57,63], *video instructions* [53], and *diaries* [52]. Users owned their diaries before engaging in the study, and their use to support learning was not planned beforehand. Nonsocial support practices were not defined after users passed the introduction phase; however, in 3% (1/31) of articles, users were encouraged to “access the video at any time during the trial” [53]. None of the articles reported the provision of only nonsocial support. Nonsocial support was always connected to social network support.

### Meanings of eHealth in Rural and Remote Areas

#### Overview

The focus of the third research question was on the meanings attached to eHealth technologies for older adults living in rural and remote areas. The terms *rural*, *remote*, and *sparsely populated area* were used to search the databases (Textbox 1) for this literature review. However, in only 55% (17/31) of articles, every participant (12/17, 71%) or part of the participants (5/17, 29%) lived in rural or remote areas. The minimum amount of rural or remote participants per selected article was 13.29%. The thematic analysis produced 17 subcategories of meanings attached to eHealth technologies for older adults living in rural and remote areas. The subcategories were clustered into 2 upper-level categories: needed for rural and remote areas (13/17, 76%) and source of inconvenience and concern (4/17, 24%). The articles defined rural and remote areas as underserved areas with limited access to health care.

#### Needed for Rural and Remote Area

Of the 17 articles, 14 (82%) reported that different eHealth technologies, such as home telehealth monitoring and videoconferencing for consultation, were needed in rural and remote areas. A total of 2 meanings clearly stood out: first, 71% (12/17) of articles [40,56] reported that eHealth technologies *facilitate access to health care services* for older rural adults; second, 65% (11/17) of articles [48,64] reported that there are *no logistical barriers* to health care services when older rural adults use eHealth technology at home. Other, less commonly reported meanings were *no temporal barriers* [61], *no financial barriers* [48]*, supports relationship with care provider* [64], *no physical or physiological stress* [39], *ability to support rural caregivers* [42], *reduces boundaries of home* [50], *reduces feelings of isolation* [44], *ensures equal access to health care services* [41], *increases feelings of security* [44], *no weather-related barriers* [39], and *permits religious or spiritual inclusion* [48].

#### Source of Inconvenience and Concern

Although eHealth technology was principally seen as needed for rural and remote areas, 24% (4/17) of articles identified sources of inconvenience and concern. Here, the most commonly coded meanings are related to internet connectivity and use. Approximately 18% (3/17) of studies reported that rural areas *lack access to high-speed internet* [40,42,61]. Approximately 12% (2/17) of studies [42,61] noted *cultural differences regarding internet use* in rural areas compared with urban areas. Older adults in rural areas are still uncomfortable using the internet. Approximately 6% (1/17) of studies [45] reported that living in rural areas required *additional equipment*, such as “protectors to protect equipment,” and rural participants had to *learn how to reset* the technology “to decrease the need for providers to make a home visit specific to technical support.” The latter was the only meaning assigned to learning to use eHealth technology in rural areas.

## Discussion

### Principal Findings

To advance the understanding of older adults’ (aged ≥60 years) eHealth learning and use during its domestication, a systematic literature review of 31 empirical studies published between 2010 and January 2020 was conducted. The aim was to summarize the literature on the barriers and enablers that older adults encounter when learning to use and using eHealth technology and how they are supported in real home settings. The main targets were rural and remote older adults. The key findings of this review confirmed that social networks supporting older adults are important enablers for learning how to use and using eHealth technology. In addition, this review revealed that health-related difficulties often prevent older adults from domesticating eHealth technologies. Various eHealth technologies have been reported as necessary for older adults in rural and remote areas, although some sources of inconvenience and concern related to internet connectivity and use were found.

One of the goals of this review was to find out which barriers and enablers are related to learning how to use and using eHealth technologies in the domestication processes among older adults living at home. The findings were divided into 4 dimensions (appropriation, objectification, incorporation, and conversion) of the domestication process [15,17]. The barriers and enablers are in line with previous similar systematic reviews [24,26]. The accessibility of support and training, usability of the technology, and its suitability for daily life were also stressed in the present review. However, for example, concerns regarding the cost or importance of motivation to use eHealth technologies were not key factors in this review. There was a significant difference between the number of reported barriers (n=48) and enablers (n=63), with enablers being more common. This may be because of a desire to highlight the benefits of eHealth technology in a study, even if unintentionally. The appropriation dimension had the most barriers, which suggests that the first phase of domestication is critical. Previous research [68,69] has expressed that access and support for older adults using technology, especially in the early weeks of its domestication, may be the most important factor in successful technology adoption. Overall, the studies focused less on the learning process than on the use of technology.

In addition, this systematic review asked how older adults living at home are supported during the domestication of eHealth technology. In most cases, older adults distributed their digital competences with their social networks, which supported the domestication of eHealth technology. The social network included *warm experts* who are “nonprofessional persons who help inexperienced users come to terms with digital devices” [70], such as family members and peers. The network also included formal personnel, such as health care professionals and technical staff. Previous qualitative research related to eHealth learning and use [71] has underlined that there is a need to clarify the role of peer-to-peer support in the domestication of technology. This study revealed that peer-to-peer support has its place in the digital health technology context [38,44,52] as well as in other settings [14]. However, health care professionals were found to be the ones with whom digital competences were most often distributed; therefore, this review argues that digital competences related to eHealth learning and use are not only technical skills but also knowledge and skills related to health care, which is why health care professionals are also needed to support eHealth technology users.

This review’s final aim was to better understand the meanings attached to eHealth technologies for older adults living in rural and remote areas. Although the literature includes few studies set in rural and remote areas, this review confirms that eHealth technologies are needed in rural and remote areas for several reasons. eHealth technology is seen as solving many problems related to limited access to health care services, such as logistical, temporal, financial, and weather-related barriers. In addition, eHealth technologies can foster a sense of belonging in older rural residents by reducing feelings of isolation and by connecting them with peers or care providers. Previous research has also shown that ICT services can reduce social isolation and promote social connectivity in older adults experiencing physical and cognitive decline or living in remote areas [3,72,73]. However, the use of eHealth technology in rural and remote areas is hampered by the lack of high-speed internet connections and older adults’ lack of comfort in using ICTs. These issues are targets for future development. The findings of this systematic review confirm that the use and nonuse of eHealth technology are related to its fit in older adults’ technology-related cultural understanding and the context in which people act and live [13,14].

This review reveals several gaps that suggest directions for future research. First, a stronger focus on older adults’ learning processes is required in research on the domestication of eHealth technology. In the literature search, numerous articles focused only on older adults and the use of eHealth technology from a biomedical [74] or technical perspective [75]. They were excluded from the review as they did not focus on the user’s point of view. Burholt and Dobbs [2] also found that research on rural aging is dominated by a biomedical perspective. Second, as this review confirmed, eHealth technology is needed in rural and remote areas. Therefore, more studies in these settings are required to better understand the processes of domesticating eHealth technology among older rural residents. Third, research efforts in eHealth technology studies in real home settings are limited. Several studies were excluded from this review as they focused on a communal dwelling [76] or nursing home [77], although the aim of the care policies is aging in place [10], which may be because it is easier to reach older adults and conduct research in organized environments. Finally, methodologically, studies on the domestication of technology were mostly qualitative, and mixed methods studies were most common [18,78]. However, the studies reviewed here were mainly quantitative. Therefore, future qualitative studies on this topic should include the voices of older adults. Furthermore, by the time this review is published, new papers relevant to this topic will have also been published. Therefore, new systematic literature reviews should be regularly conducted on the topic of eHealth knowledge and use among older adults living at home in rural and remote areas.

### Limitations

This review has several limitations. First, as there was only 1 reviewer involved, this review’s quality and reliability were somewhat weakened. Additional reviewers would have challenged the decisions made by the author. Second, although the selected studies were conducted in 12 countries, Western countries and English-speaking countries dominated, and studies not written in English were excluded. As noted in earlier reviews [30], this may create a bias by, for example, basing the findings mainly on reports from Western cultures. Furthermore, despite this review’s intent, not all the studies were set in rural and remote areas, and therefore, its findings cannot be directly adopted in rural and remote contexts, with the exception of those of the third research question. The search included a comprehensive variation of health- and technology-related search terms (Textbox 1). The search terms did not include common abbreviations, such as *eHealth* and *mHealth.* However, these absences did not likely have a significant effect on the results. In addition, health technologies develop rapidly [35]; therefore, this review’s start date of 2010 may seem distant. However, a 10-year period is commonly used in qualitative reviews of eHealth technology [14,29]. As noted by Vuojärvi [79], “although technologies change rapidly, people and the ways technologies are used in everyday lives, and particularly in the learning process, do not necessarily do so.”

### Conclusions

This literature review provides information and practical implications for designers, health care providers, and policy makers. eHealth technology targeted at older adults should be easy to use, and adequate support and training should be provided to users [25,80]. There are plenty of barriers that prevent older adults from ably and independently using eHealth technologies at home. Special attention should be paid to the most common barriers to learning how to use and using eHealth technologies: health-related difficulties, lack of internet connectivity, and other technical problems. We would like to emphasize the importance of considering older adults’ social and cultural practices when designing and implementing eHealth technologies. Social network support and technology integration into everyday life in rural areas contribute to a successful domestication process.

## Appendix

Multimedia Appendix 1 PRISMA (Preferred Reporting Items for Systematic Reviews and Meta-Analyses), a 27-item checklist.

Multimedia Appendix 2 An example search string for Scopus (Elsevier).

Multimedia Appendix 3 Summary of included articles.

## Abbreviations

ICT: information and communication technology
PRISMA: Preferred Reporting Items for Systematic Reviews and Meta-Analyses
