# Supplementary material for: Learning and Use of eHealth Among Older Adults Living at Home in Rural and Nonrural Settings: Systematic Review
Source: J Med Internet Res. 2021 Dec 2;23(12):e23804. doi: 10.2196/23804 (PMC8686468; doi:10.2196/23804)
Supplement: Multimedia Appendix 2 [file jmir_v23i12e23804_app2.pdf]

Multimedia Appendix 2. An example search string for Scopus (Elsevier).

TITLE-ABS-KEY (*older* OR *senior* OR *elderly* OR *aged* OR *old* AND *age* AND *user* OR *elder*) AND (*rural* OR *remote* OR *sparsely*) AND (*online* OR *ict* OR *information* OR *computer* OR *internet* OR *electronic* OR *technolog** OR *digital* OR *smart* OR *"management* AND *tool"* OR *virtual* OR *mobile* OR *robot* OR *tele** OR *monitoring* OR *assist** OR *gerontechnology* OR *compliance* OR *reminder* OR *dispens** OR *video* OR *application* OR *device*) AND (*use* OR *education* OR *learn** OR *competence* OR *"digital* AND *skill"* OR *geragogy* OR *reject* OR *"active* AND *aging"* OR *adoption* OR *acceptance* OR *barrier* OR *enabler* OR *facilitator*) AND (*health* OR *care* OR *wellbeing* OR *physical* OR *mental* OR *social*) AND (*"aging* AND *in* AND *place"* OR *independent* OR *home* OR *"everyday* AND *life"* OR *living* OR *"daily* AND *life"* OR *domestication*) AND (LIMIT-TO (PUBYEAR , *2019*) OR LIMIT-TO (PUBYEAR , *2018*) OR LIMIT-TO (PUBYEAR , *2017*) OR LIMIT-TO (PUBYEAR , *2016*) OR LIMIT-TO (PUBYEAR , *2015*) OR LIMIT-TO (PUBYEAR , *2014*) OR LIMIT-TO (PUBYEAR , *2013*) OR LIMIT-TO (PUBYEAR , *2012*) OR LIMIT-TO (PUBYEAR , *2011*) OR LIMIT-TO (PUBYEAR , *2010*)) AND (LIMIT-TO (DOCTYPE , *"ar"*)) AND (LIMIT-TO (LANGUAGE , *"English"*))
