# Supplementary material for: Learning and Use of eHealth Among Older Adults Living at Home in Rural and Nonrural Settings: Systematic Review
Source: J Med Internet Res. 2021 Dec 2;23(12):e23804. doi: 10.2196/23804 (PMC8686468; doi:10.2196/23804)
Supplement: Multimedia Appendix 3 [file jmir_v23i12e23804_app3.pdf]

Multimedia Appendix 3. Summary of included articles.

Article	*Method*	*eHealth technology/target group*	*Number, gender, and age of older participants*	*Older participants country/living area*
Baharin et al. (2015) [52]	Qualitative (ethnographic; interviews, diaries, observations) Quantitative (technical logs)	Ambient awareness technology, interaction radio / independently living seniors	n = 3 dyads (0% male) Ages of dyads: 67 and 79, 67 and 60, 70, and “in her 30s”	Remote location
Barrera et al. (2017) [48]	Quantitative (self-report measures), Qualitative (interviews)	Technology-delivered (telephone) cognitive behavioral therapy / older adults with depression or anxiety	n = 3 (100% male) Ages: 62, 66, 67 years	USA, rural
Blusi et al. (2015) [50]	Qualitative (interviews)	Internet-based support service / older family carers	n = 31 (25.8% male) Median age: 75 Age range: 65–85 years	Northern Sweden, rural area
Blusi et al. (2013) [42]	Qualitative (webcam-interviews)	ICT based support service / older family carers	n = 31 interviewees (25.8% male) Median age: 75 Age range: 65–85 years	Northern Sweden, vast rural areas
Bock et al. (2016) [75]	Mixed methods (feedback from experts, online survey)	A smart home monitoring system, consisted of sensors / older adults	n = 19 (36.8% male) of which n = 7 were older adult participants, at least 60 years old	Not defined
Boquete et al. (2011) [35]	Quantitative (stress test, survey)	A cognitive training program via television / elderly	n = 6 Average age: 67.83 (SD 4.07) Age range: 61–72 years	Not defined
Colón-Semenza et al (2018) [36]	Mixed methods (measures, technical logs, questionnaires, focus group)	mHealth targeting physical activity /people with Parkinson’s disease	n = 10 (peer coaches n = 5, peer mentees n = 5), (60% male) Coaches age: 64.6 (SD 4.04) Mentees age: 63.4 (SD 2.06)	USA
Costa et al. (2017). [59]	Quantitative (interaction data, questionnaires)	Interactive TV-based game platform / cognitive evaluation of senior adults	n = 62 (50% male) Age range: 65–90+	Spain, Region of Galicia, rural residents 45.16%

Evangelista et al. (2015)<br>[64]	Quantitative, (clinical information, self-reported measures, telephone surveys)	Remote monitoring system / older chronic heart failure patients	n = 21 (47.6% male)<br>Mean age: 72.7 years (SD 8.9)<br>Age range: 58–83 years	USA, Southern California
Far et al. (2015)<br>[57]	Quantitative (self-reports, attrition rate, technical logs, system usability scale)	Virtual gym on tablet / older people	n = 37 (24.3% male)<br>Age range: 65–87	Italy, Trento
Garattini et al. (2012)<br>[53]	Qualitative (individual and focus group interviews, checkpoint calls), Quantitative (questionnaire, system use measures)	Communication technology, a touch screen, designed to support social interaction / lonely older people	n = 19 (36.8% male)<br>Age range: 65–84 years	Ireland, Dublin or surrounding areas
González de Garibay et al. (2016)<br>[60]	Quantitative (questionnaire, technical logs)	mHealth app for self-management and education / patients with cardiac diseases	n = 32 (50% male)<br>58.63 (SD 18.45),<br>patients older or younger than 60 years old were equally represented	Spain, Province of Valladolid,<br>patients living in urban or rural areas were equally represented
Hicken et al. (2017)<br>[40]	Quantitative (interview short-forms, questionnaire)	Telehealth vs. Internet and phone, support for caregivers / caregivers of veterans with dementia	n = 231 (10% male)<br>70.16 years (SD 11.22)	USA,<br>rural residents 53%
Holz et al. (2015)<br>[55]	Quantitative (self-reported measures, technical logs)	Brain–computer interface, “Brain Painting” / patient with amyotrophic lateral sclerosis (ALS) in the locked-in state	n = 1 (0% male)<br>73 years old	Germany
Luptak et al. (2010)<br>[49]	Quantitative (attrition rate, survey)	Care coordination home telehealth monitoring /older rural veterans	n = 132 (90.9% male)<br>age/n<br><50/1<br>50–59/16<br>60–69/20<br>70–79/50<br>80–89/44<br>90–99/44	USA,<br>rural and remote areas

Naik et al. (2012)<br>[56]	Quantitative (medical records, surveys, interviews and session attendance data), Qualitative (case study reports)	Telephone-delivered health coaching / rural-living older adults with uncontrolled diabetes and depressive symptoms	n = 8 (86% male)<br>Mean age: 62 years<br>Age range: 58–67 years	Rural
Ottenberg et al. (2013)<br>[51]	Qualitative (focus group interviews)	Remote monitoring / patients living with an implantable cardioverter-defibrillator	n = 9 (66.7% male)<br>Median age: 73 years<br>Age range: 58–91 years	USA, Minnesota, Olmsted County
Parker et al. (2015)<br>[61]	Quantitative (online questionnaire)	Internet-based platform / patients who have received radiation treatment for prostate cancer	n = 358 (100% male)<br>Mean age: 73 (SD 8)<br>aged 60 years or older n = 219<br>younger than 60 years n = 139	Canada,<br>rural area: n = 96 (27%)
Pavic et al. (2019)<br>[41]	Quantitative (sensor data and other technical logs, self-reports, questionnaires, number of emergency visits, readmissions, deaths), Qualitative (interviews)	Remote monitoring using wearables / cancer patients in palliative care	n = 31 (71% male)<br>Median age: 64 years<br>Age range: 53–71 years	Switzerland, Zurich
Philip et al. (2015)<br>[38]	Second stage of the research: Qualitative (observations, interviews)	online pain management program / older rural population with chronic pain	n = 7 nonuser observations and interviews, age range: 60-75 years<br>n = 4 older user interviews	UK, Scotland, rural and remote areas
Piau et al. (2019)<br>[58]	Quantitative (health records, app usage, user feedback)	smartphone chatbot / cancer patients	n = 9 (55.6% male)<br>Mean age: 83.4 years (SD 2.1)	France
Pigini et al. (2017)<br>[44]	Mixed methods (technical logs, comments, questionnaire)	Home telemonitoring sensors and wearable / elderly people with cardiac diseases	n = 13<br>Aged range: 53–81 years	Italy, Milano
Read Paul et al. (2019)<br>[37]	Quantitative (questionnaire), Qualitative (interviews, focus groups, open-	Web-based videoconferencing / rural elderly palliative patients	n = 10<br>Mean age: 77 years	Canada, Alberta, rural area

	ended questions on questionnaire)			
Sadek et al. (2018) [54]	Quantitative (survey, technical logs)	Remote monitoring of sleep, sleep mat	n = 3 (0% male)<br>Ages: 65, 68, 69 years	Not defined
Saleh et al. (2018) [62]	Quantitative (survey), Qualitative (focus group)	mHealth (SMS) / noncommunicable diseases (hypertension and/or diabetes)	n = 606 (47.9% male)<br>Age range: 40–76+ years of which 69.7% were over age of 50	Lebanon, rural settings and Palestinian refugee camps
Sorocco et al. (2013) [43]	Mixed methods (satisfaction and interview data)	Care coordination home telehealth and telemental health / veterans with complex medical condition	n = 6<br>Mean age: 71.83 years (SD 10.6)	USA, Rural Oklahoma
Strain et al. (2019) [63]	Quantitative (survey)	Smartphone apps and wearable activity trackers / weight management	Sample 1<br>n = 2639 (48.8% male)<br>Age reange:16–65+ years of which 19.35% were over age of 65<br><br>Sample 2<br>n = 4380 (49.8% male)<br>Age range: 16–65+ years of which 21.9% were over age of 65	UK<br>Sample 1<br>Rural residents 13,29%<br><br>Sample 2<br>Not defined
Vaskinn et al. (2013) [39]	Quantitative (interview, questionnaires)	Telephone-based screening service / elderly people with mild cognitive impairment	n = 39 (71.8% male)<br>Median age: 70 years<br>Age range: 61–91 years	Rural northern Norway, Bardu
Wakefield et al. (2014) [47]	Quantitative (medical records, interviews, questionnaires)	Remote telephone-based rehabilitation program / cardiac rehabilitation	n = 43 (98% male)<br>Mean age: 63.7 (SD 8.2)	USA
West et al. (2010) [65]	Quantitative (goal-setting protocol)	Home telemedicine unit, that is, a Web-enabled computer used to videoconference / older adults with diabetes	n = 610 (44.9% male)<br>all >55 years of age,<br>85.6% over 65 years old	USA, rural upstate New York

Williams et al. (2016) [45]	Quantitative (system usability scale, video recorded observations)	Telemonitoring application / older people	n = 7 (71.5% male)<br>Age range: 70–86 years	Not defined
